# Solvent-Free Synthesis of Phosphonic Graphene Derivative and Its Application in Mercury Ions Adsorption

**DOI:** 10.3390/nano9040485

**Published:** 2019-03-27

**Authors:** Robert Olszewski, Małgorzata Nadolska, Marcin Łapiński, Marta Prześniak-Welenc, Bartłomiej Michał Cieślik, Kamila Żelechowska

**Affiliations:** 1 Department of Solid State Physics, Faculty of Applied Physics and Mathematics, Gdansk University of Technology, Narutowicza 11/12, 80-233 Gdansk, Poland; robercik.olszewski@gmail.com (R.O.); malgorzata.nadolska@pg.edu.pl (M.N.); marcin.lapinski@pg.edu.pl (M.Ł.); marta.welenc@pg.edu.pl (M.P.-W.); 2 Faculty of Chemistry, Department of Analytical Chemistry, Gdansk University of Technology, Narutowicza St. 11/12, 80-233 Gdansk, Poland; cieslik1988@wp.pl

**Keywords:** graphene derivatives, mechanochemical synthesis, ball mill synthesis, mercury adsorption

## Abstract

Functionalized graphene was efficiently prepared through ball-milling of graphite in the presence of dry ice. In this way, oxygen functional groups were introduced into material. The material was further chemically functionalized to produce graphene derivative with phosphonic groups. The obtained materials were characterized by spectroscopic and microscopic methods, along with thermogravimetric analysis. The newly developed material was used as an efficient mercury adsorbent, showing high adsorption efficiency. The adsorption isotherms were fitted using Freundlich and Langmuir models. The adsorption kinetics were fitted with pseudo-first order and pseudo-second order models. Adsorption selectivity was determined in the presence of cadmium ions and nickel ions. The presence of mentioned bivalent ions in the solution did not affect mercury adsorption efficiency.

## 1. Introduction

Graphene with its multifunctionality attracts interest in many fields of science [1]. It is characterized by unique chemical and physical properties such as high mechanical strength, elasticity, high thermal conductivity, and high electron mobility [2,3,4]. Not only are basic studies devoted to graphene carried out, but it has also become the main subject of research in many fields of engineering. The number of potential applications of graphene is remarkable. Graphene and its derivatives are considered to be useful in polymer fuel cells [5], solar cells [6], field effect transistors [7], sensors [8], optoelectronics [9], composites and many others [10]. Moreover, functionalization of graphene is an effective way to alter its physico-chemical properties, widening its applications spectrum [11,12]. With the rapid development of science and industry, the demand for functionalized graphene is growing and it is necessary to develop an effective and convenient scalable method to prepare high quality graphene derivatives.

The aim of this study is to develop low-cost and effective method of phosphonic graphene derivatives’ production with tailored properties. Incorporation of phosphonic functional groups is a commonly used approach in surface modification [13] and is often applied to enhance materials chemical [14] and flame [15,16] resistance or biocompatibility [17,18]. In addition, phosphonic -based materials show excellent complexing properties and can interact with a wide range of metal ions [19,20]. However, in the literature, phoshponic graphene derivatives have rarely been reported and only a few works in this subject can be found [21,22,23,24,25]. Ball-milling is a simple and effective, low-cost method for production of carbon nanoplatelets and allows for producing graphene-derivatives on a large scale. However, only a few research works have been conducted in this area [26,27,28,29,30,31]. Synthesis of graphene and its derivatives in gaseous phase, as a widely used chemical vapour deposition method, suffers from costly reagents and usage of specialized equipment. Beneficially, the graphene produced, is of high quality, which is appropriate for applications in electronics. However, for some applications, the obtained material does not have to possess an ideal structure. Moreover, chemical functionalization, which purposely distracts the hexagonal lattice of graphene is frequently implemented in order to tailor physicochemical properties of this nanostructure. It is especially well visible in applications connected with interfacial interactions, as, for example, sorption studies. Graphene oxide (GO) which may be concerned as an oxidized form of graphene is successfully used as an adsorbent for different pollutants, including dyes or metal ions [32,33]. However, the synthesis of GO is performed under harsh conditions, with the usage of strong acids and highly toxic manganese compounds. After synthesis, the copious amount of water is used in order to purify the obtained material [34,35,36,37]. Taking it into account, the proposed protocol for GO synthesis is far from the “green” approach. Overall, the benefits from GO usage are hindered by its burden to the environment synthesis.

Here, we propose an efficient and low emission method for functionalized graphene derivative manufacturing. Graphite was ball-milled under solvent free conditions in the presence of solid CO_2_. The obtained graphene derivate with introduced oxygen groups (mainly carboxylic groups) was further reacted with PCl_3_/H_2_O mixture in order to convert -COOH groups to bisphosphonic groups. It is an industrially known reaction, used in the synthesis of pharmaceuticals [38,39,40,41,42]. However, in our approach, we eliminated the usage of solvents and the reagents served as the reaction medium. It is also worth noting that the unreacted PCl_3_ can be distilled off after synthesis and redirected into the next reaction. Such approach ensures the maximal usage of the reagents, making the whole procedure more sustainable.

The obtained graphene derivative with phosphorus bearing groups was proposed as an efficient adsorbent for mercury ion removal from water. The phosphonic and bisphosphonic groups show high affinity to mercury ions, thus the adsorption capacity and selectivity were expected to be substantially improved after introduction of such groups into the graphene derivative. Moreover, next to the functional groups, the defects created during ball-milling can play a crucial role in the adsorption process. It is well known that defects in the adsorbent can act as active sites for adsorbate anchoring. Therefore, the obtained material possesses a promising structure for proposed application.

## 2. Materials and Methods

### 2.1. Materials

Phosphorus trichloride (PCl_3_, Merck, Darmstadt, Gemrany, Purity >99%) was used without any further purification. Carbon dioxide, graphite flakes (+100 mesh, >75%, product number 332461) and HgCl_2_ were purchased in Sigma-Aldrich (Poznan, Poland). Filter paper no. 06-0014 was purchased in Chemland (Stargard Szczecinski, Poland).

### 2.2. Material Preparation

A typical experiment was carried out in a planetary ball-mill (Pulverisette 6, Fritch, Idar-Oberstein, Germany), using zirconium oxide containers and balls. The synthesis was conducted in the presence of dry ice (40 g) and 2 g of pristine graphite (the mass ratio 20:1) and 40 zirconium oxide balls. The rotational speed of the mill was 350 RPM, and the milling time was 48 h with 10 min pauses on each 50 min of milling. The obtained graphene derivative with oxygen groups was denoted as GCO2. In the next step of the experiment, GCO2 sample was reacted with phosphorus trichloride in water in order to introduce bis-phosphonic groups into the carbonaceous structure. After 5 h of stirring at 50 °C, the residual liquids were decanted and the solids were suspended in 20 mL of water. The mixture was heated under reflux for 1 h; then, the sample was separated by centrifugation and the solids were washed with water until pH reached a value of 7.0 ± 0.2. The obtained product, denoted as GPhos, was dried at 40 °C under reduced pressure (0.1 bar).

Another material—milled graphite (GM)—was also obtained by a ball-milling method. The milling process was carried in a planetary ball-mill and it was conducted under the same conditions with the exception that 2 g of pristine graphite was milled, with no other reagents. Milling of pure graphite was performed to investigate the difference in the material structure between milled graphite (GM) and graphite after ball-milling with dry ice (GCO2). Therefore, the GM sample served as a reference for all studies.

### 2.3. Research Methodology

The Fourier Transformed Infrared (FTIR) spectra were recorded using a KBr pellet method on a PerkinElmer Frontier spectrophotometer (Waltham, MA, USA) with a resolution of 2 cm^−1^ in the range of 4000 cm^−1^–500 cm^−1^—number of accumulations: 10 times each. KBr was mixed with tested powder in such quantities to get a transmission in the range of 20% to 70%.

Raman spectra were recorded using a Renishaw InVia spectroscope (Renishaw, New Mills, UK) with argon ion laser operating at 514.5 nm focused through a 50× objective. The laser power was reduced to 5% of maximum power at 514 nm to avoid sample damage. The spectra were collected in the dark, with resolution of 2 cm^−1^ in the range of 100–3200 cm^−1^ and 3 accumulations. For analysis, each peak was fitted using Origin Pro 9.1 (OriginLab Corporation, Northampton, MA, USA) with user defined initialization parameters. Lorentzian fits were characterized by much better R^2^ parameter than Gaussian fits. All fits achieved R^2^ greater than 0.98.

X-ray Photoelectron Spectroscopy (XPS) analyses was carried out with an Omicron NanoTechnology X-ray photoelectron spectrometer (Scienta Omicron GmbH, Taunusstein, Germany) with a 128-channel collector. The measurements was performed at room temperature in ultra-high vacuum conditions. The photoelectrons was excited by an Mg-K X-Ray source. An Omicron Argus hemispherical electron analyser with round aperture of 4 mm is used for analysing of emitted photoelectrons. The binding energies are calibrated to obtain C=C peak at 284.5 eV and C-C at 285.2 eV. XPS spectra were analysed with Casa-XPS software (Casa Software Ltd, Teignmouth, UK) using a Shirley background subtraction and Gaussian–Lorentzian curve as a fitting algorithm.

Thermogravimetric analysis (TGA) was performed under argon atmosphere from 40 °C to 900 °C at a linear heating rate 10 °C/min using Netzsch STA 449 F1 (Netzsch, Selb, Germany). To avoid heat and mass transfer limitations, approximately 10 mg of the sample was used, and Al_2_O_3_ crucibles with lids were employed.

The morphology of nanomaterials was studied using scanning electron microscopy (ESEM Quanta Feg 250, FEI, Thermo Fisher Scientific, Waltham, MA, USA). The elemental analysis was performed by energy-dispersive X-ray spectroscopy (EDX) using the EDAX Genesis APEX 2i with Apollo X SDD spectrometer (EDAX Inc., Mahwah, NJ, USA) at 10 kV.

Specific surface area (SSA) was determined by a Brunauer–Emmett–Teller (BET) method from nitrogen adsorption–desorption isotherms (77 K, surface area analyser NOVAtouch™ 2, Quantachrome Instruments, Boynton Beach, FL, USA). In all calculations, the correlation coefficient for the linear BET plot was at least 0.9998. Before the measurements, samples were degassed under vacuum at 40 °C for 12 h.

Adsorption studies of Hg^2+^ ions were carried out in the batch mode at room temperature and pH 7, without stirring or shaking. In order to study adsorption kinetics and isotherms, measurements were performed using different contact time, Hg^2+^ ion concentration and amount of adsorbent. Solutions with desired Hg^2+^ ion concentration were obtained from the dilution of the stock solution which was prepared by dissolving HgCl_2_ in Milli-Q deionized water. After appropriate contact time, adsorbents were separated from the metal ion solutions through a filter paper (Chemland, no. 06-0014). Concentration of Hg^2+^ in the solutions before and after adsorption were determined by atomic absorption spectrometry (AAS, GBC SCIENTIFIC EQUIPMENT, Hampshire, IL, USA). Adsorption efficiency was then calculated according to the typical equation (Equation (1)):

(1) $$Adsorption\;efficency\left(\%\right)=\frac{C_{0}-C_{t}}{C_{0}}\times 100\%,$$

where *C*_0_ (mg/L) is the initial Hg^2+^ ion concentration and *C_t_* (mg^−1^) is the concentration of Hg^2+^ ion after appropriate contact time.

Two kinetic models: pseudo-first order (Equation (2)), pseudo-second order (Equation (3)) were applied to understand the adsorption dynamics of Hg^2+^ on GPhos. The kinetic studies were carried out using 1 mg of GPhos, 20 mL of Hg^2+^ solutions (1 mg/L) and contact time varying from 0.5–21 h.

Pseudo first-order equation:

(2) $$\ln\left(q_{e}-q_{t}\right)=lnq_{e}-k_{1}t,$$

Pseudo second-order equation:

(3) $$\frac{t}{q_{t}}=\frac{1}{k_{2}q_{e}^{2}}+\left(\frac{1}{q_{e}}\right)\times t,$$

where, *q_e_* and *q_t_* (mg/g) represent the amount of metal ions adsorbed at equilibrium and time *t*. *k*_1_ (min^−1^) and *k*_2_ (min·g/mg) are the pseudo-first-order and pseudo second order rate constants, respectively [43].

To determine the type of adsorption and the capacity of the GPhos, Langmuir and Freundlich models were applied. In these studies, 1 mg of GPhos was added into 20 mL solutions. The contact time was set for 21 h and the Hg^2+^ concentration was: 0.5, 1.0, 1.5, 3.0, 5.0, 8.0, 10.0 and 16.0 mg/L. The Langmuir isotherm model is based on a monolayer adsorption of metal ions onto a homogenous surface. It assumes equivalent sorption energies and no interaction between adsorbed species. The linear form of this isotherm is represented by the expression:

(4) $$\frac{C_{e}}{q_{e}}=\frac{C_{e}}{q_{m}}+\frac{1}{bq_{m}},$$

where *C_e_* (mg/L) is the equilibrium concentration of metal ions, *qe* (mg/g) signify the concentration of metal ions adsorbed per unit mass of the adsorbent and *q_m_* (mg/g) and *b* (L/mg) are Langmuir constants that indicate the maximum monolayer adsorption capacity and energy constant related to the affinity of the binding sites, respectively. In comparison, the Freundlich isotherm model explains the interaction between adsorbate molecules and adsorbents with multilayer adsorption on heterogeneous surfaces. The linear form of Freundlich isotherm is given by Equation (5):

(5) $$lnq_{e}=\ln K+\frac{1}{n}\times \ln\left(C_{e}\right),$$

where *K* (L/g) and *n* (g/L) are Freundlich constants corresponding to adsorption capacity and adsorption intensity, respectively [44].

## 3. Results

### 3.1. Synthesis

**Figure 1:**
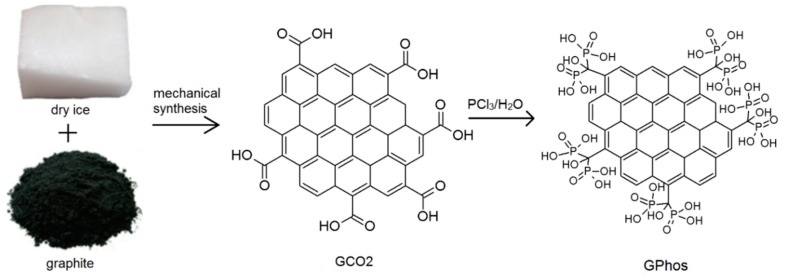
Schematic representation of the mechanochemical synthesis.

Milling of graphite with solid CO_2_ in a ball-mill was expected to introduce the oxygen functionalities and delaminate functionalized graphite platelets. Then, the functionalized product was subjected to the reaction with phosphorus trichloride in water environment. The reaction is a conversion of carboxylic groups (-COOH) into respective bis-phosphonic groups. The mechanism of the reaction was described elsewhere, and we successfully used this approach in carbon nanotubes and graphene oxide functionalization [21,45]. Schematic representation of the mechanochemical synthesis is shown in Figure 1.

The proposed approach benefits from the elimination of solvent in the first step of the reaction. Moreover, no by-products are formed and there is no need to purify the obtained material; thus, it can be directly used in the next step of the reaction. The chemical conversion of GCO2 into GPhos is a one-pot synthesis, with usage of cheap and available reactants. The unreacted PCl_3_ can be redirected into the next reaction, making the whole procedure more efficient. Therefore, it can be easily scaled-up and used for mass production of graphene derivatives. In order to confirm the successful chemical functionalization spectroscopic, microscopic and thermal analyses were performed.

### 3.2. Raman Spectroscopy

**Figure 2:**
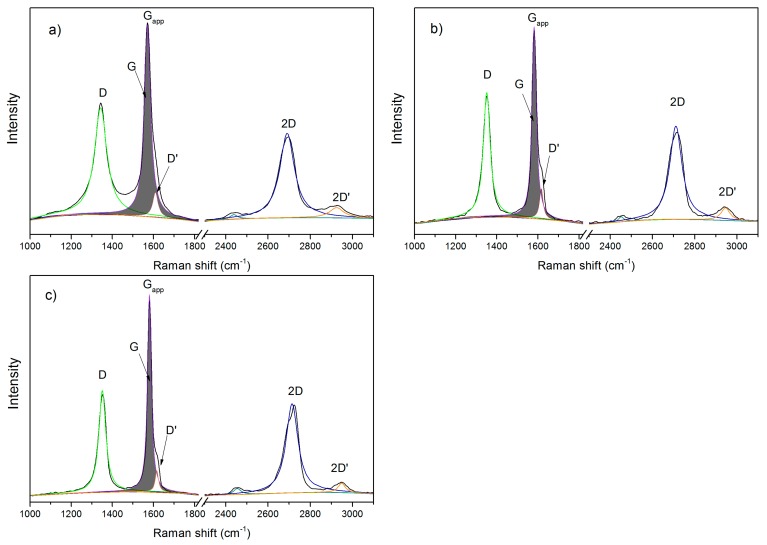
Raman spectra for (**a**) GM; (**b**) GCO2; (**c**) GPhos.

Raman spectroscopy was used to characterize the obtained materials. The results are presented in Figure 2 and in Table 1. The ball-milling of graphite with CO_2_ was expected to introduce oxygen groups into edges of graphene planes, making the structure similar to reduced graphene oxide (rGO). For comparison, the Raman spectra of GM were also recorded. It was carried out in order to verify if the structural changes are only due to mechanical cracking of the material or due to the functional groups’ introduction. The analysis of Raman spectra was carried out in accordance with the latest theory proposed by King et al. [46]. It assumes that the classic analysis of Raman spectra by isolating D, G and 2D bands is insufficient for carbon nanostructures. According to this theory, it is possible to extract the G and D’ mode from the G_app_ band and 2D’ mode. As can be seen in Figure 2 bands G and 2D have been subjected to deconvolution that resulted in bands G and D’ and 2D and 2D’, respectively. The positions of D and G bands for analyzed samples are compared in Table 1. 

In the case of material with introduced oxygen groups (GCO2), the G mode is shifted 10 cm^−1^ to higher wavenumbers in a relation to G mode for GM. It may indicate that the number of defects is lower for GCO2 sample. Moreover, the D’ band is present in all defective graphenes and it is great as a measure of amount of defects. In the case of the GM sample, the D’ peak is higher than for GCO2 and GPhos samples. This may indicate a more degraded structure. During the ball-milling of graphite in the presence of air, the sample is partially destroyed due to the high temperature occurring locally and possibility of the combustion. It can be seen by the naked eye that the GM sample is rather in a powder form, with possibly a high degree of amorphous carbon. Such assumption was further made evident by a microscope in a thermogravimetric analysis. Taking into account that CO_2_ is used as an extinguishing substance, it acts in the reaction as a combustion suppressant, preventing the sample from uncontrollable destruction. Thus, in contrast to GM, the GCO2 sample remained its layered structure with a lower number of defects in the hexagonal plane, which was confirmed by a microscopy and Raman analysis.

In all cases, band D is lower than appropriate band G. In addition, the intensity ratio of I_D_/I_G_, the full width at half maximum of D’ and the difference between 2D’ and G_app_ were analysed (Table 1). The analysis confirms the introduction of functional groups into the material, as made evident by changes in all the listed parameters. As can be seen in Table 1, differences between GCO2 and GPhos are less distinct, which is reasonable. The reaction with PCl_3_/H_2_O does not lead to structural changes of material, except for replacing the carboxyl groups by bis-phosphonic groups. Therefore, no substantial differences in Raman spectra of GCO2 and GPhos samples are expected. The presence of phosphonic groups was made evident by other methods (FTIR, XPS).

### 3.3. FTIR Spectroscopy Results

**Figure 3:**
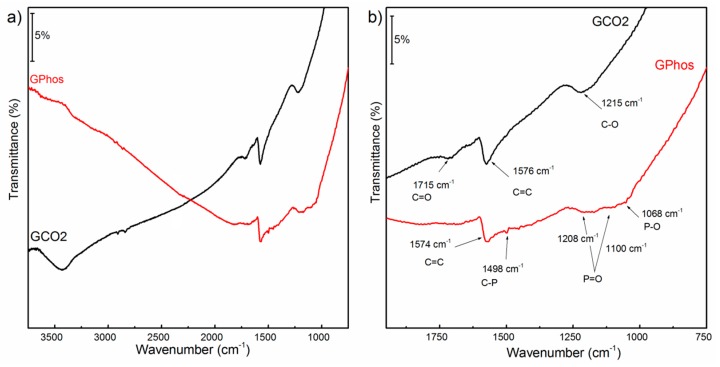
Fourier Transformed Infrared spectra of GCO2 and GPhos (**a**) the mid-IR range spectra; (**b**) zoomed over 750–1800 cm^−1^.

The FTIR spectroscopy was used to determine the functional groups present in the samples. The spectra recorded for GCO2 and GPhos samples are presented in Figure 3a. Figure 3b shows a zoomed region of 750–1800 cm^−1^, with band assignment. Both spectra show bands at c.a. 1576 cm^−1^, corresponding to C=C vibrations in graphene planes. The FTIR spectrum of a GCO2 sample (Figure 3a,b) reveals bands at 3430 cm^−1^, 1715 cm^−1^ and 1215 cm^−1^, which can be ascribed to -OH, C=O and C-O bonds, confirming the presence of carboxyl groups in the sample. In the spectrum registered for GPhosthe, a 3430 cm^−1^ band disappeared; however, new bands can be observed due to the presence of phosphonic groups. The multiple band in the region 1300–1000 cm^−1^ confirms the presence of P=O and P-O bonds in the analyzed structure. A weak band at 1498 cm^−1^ originating from C-P stretching can also be observed. The disappearance of bands ascribed to -COOH groups is an additional piece of evidence of successful conversion of the carboxylic groups into phosphonic ones [21,45].

### 3.4. XPS Spectroscopy Results

To further verify results obtained from FTIR analysis, GCO2 and GPhos were characterized by XPS. As expected, the wide XPS survey spectrum of GCO2 (SI, Figure S1) revealed that the surface of this sample consists of only C and O atoms. After reaction with PCl_3_, in the GPhos spectrum, a new signal at ca. 134 eV can be observed, which confirmed the presence of P atoms on the sample surface (SI, Figure S2). Table 2 summarizes the atomic percentage of elements on GCO2 and GPhos. The analysis showed that the materials consist mainly of carbon. Taking into account the assumed mechanism of the reaction, it can be concluded that functional groups are mainly located at the edges of carbon flakes. Therefore, the number of functional groups in relation to carbon atoms building the graphitic skeleton is comparatively low and, thus, the oxygen and phosphorus contents are also low.

**Figure 4:**
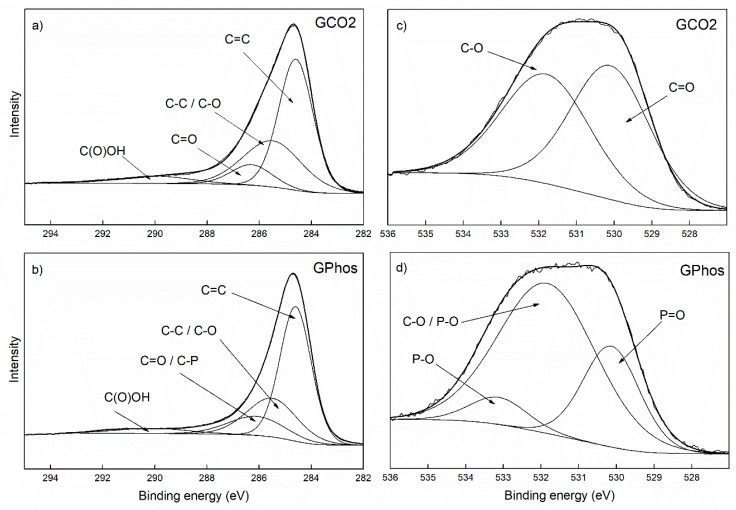
Deconovoluted C1s (**a**,**b**) and O1s (**c**,**d**) high resolution X-ray Photoelectron Spectroscopy spectra for GCO2 and GPhos.

In order to determine the chemical state of atoms in the analyzed samples, the C 1s and O 1s bands were deconvoluted (Figure 4). The C 1s spectrum for GCO2 was divided into four components: C=C (284.5 eV), C-C/C-O (285.2 eV), C=O (286.3 eV) and C(O)OH (290.0 eV) [47,48]. The O 1s band was divided into two peaks C-O (531.4 eV) and C=O (529.7 eV), proving the presence of oxygen groups in the GCO2 sample as an effect of reaction with dry ice under ball-milling. The C 1s spectrum for GPhos was divided into four parts C=C (284.5 eV), C-C/C-O (285.2 eV), C=O/C-P (286.1 eV) and a small peak at ca. 290.0 eV. The O 1s peak was divided into three parts (533.1, 531.8, 530.1 eV), which represent chemical bonds (on the order of decreasing binding energy) P-O, C-O/P-O and P=O, respectively (Figure 4b,d). The presence of C-P and P-O/P=O components in adequate bands confirmed the expected structure of a GPhos sample. Moreover, the XPS results are consistent with FTIR spectroscopy, both proving the presence of phosphonic groups.

### 3.5. SEM Analysis

**Figure 5:**
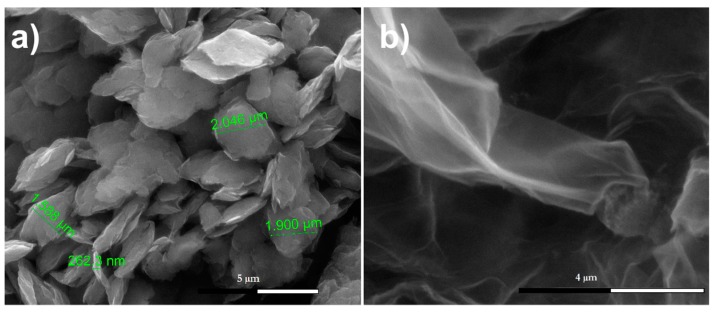
SEM images of GPhos sample (**a**) at a magnification 10,000; (**b**) at a magnification 50,000.

An SEM technique was used to demonstrate the mechanochemical cracking of large flakes of graphite into a small grain size present in the GPhos sample. The pristine graphite flakes were close to 50 µm in size. After ball-milling for 48 h and chemical reaction, the resultant GPhos shows a dramatically reduced size of flakes, which were in the range from 100 nm to thousands of nanometers (Figure 5a,b). Higher magnifications revealed the presence of exfoliated sheets made of a few carbon layers (Figure 5b). The EDX measurement was carried out for 10 points on the sample surface. As expected, the results show the presence of constituent elements for sample GPhos. High content of oxygen (33.2% atomic, 55.3% mass), carbon (66.3% atomic, 39.5% mass) and the presence of phosphorus (0.5% atomic, 1.12% mass) was observed. The results are in agreement with XPS analysis. However, in contrast to XPS measurements, the EDX analysis is performed under a low vacuum (10^−2^ mbar), which is not sufficient for removal of adsorbed gases from the sample surface. Therefore, higher oxygen content was determined by EDX.

### 3.6. TGA Analysis 

**Figure 6:**
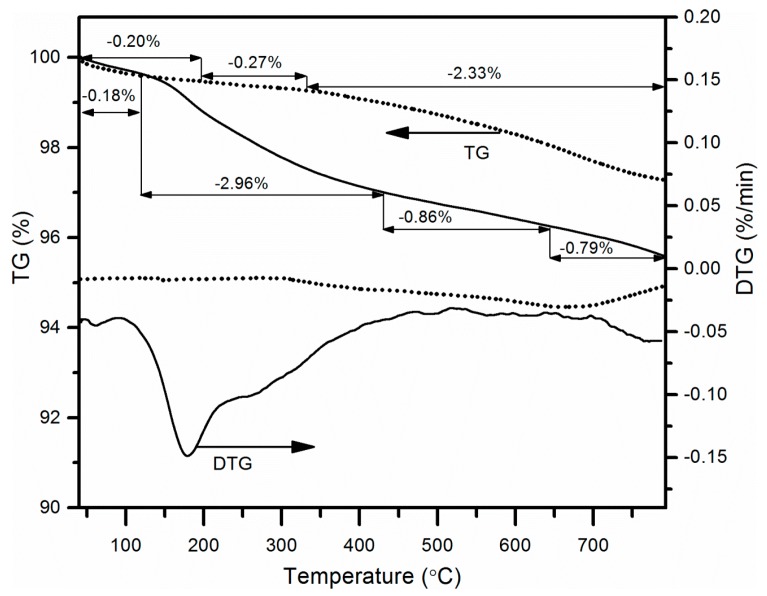
Thermal gravimetric analysis of GCO2 and GPhos samples.

Thermal stability of the materials was assessed by thermogravimetric analysis in argon (Figure 6). For all analysed samples, decomposition was observed—however, to a varying degree. The GCO2 sample lost about 3% and GPhos lost about 5% of initial mass. According to the literature, the mass loss up to 180 °C is due to the desorption of molecules, mainly water from the material surface. For functionalized carbon materials, further mass loss in a higher temperature range can be ascribed to functional groups’ degradation [49]. As can be seen in Figure 6, GCO2 and GPhos samples behave similarly in temperatures up to 180 °C, with a more substantial mass loss observed for a GPhos sample in the higher temperature range. Taking into account the higher molar mass of bis-phosphonic group (in GPhos sample) as compared to a carboxylic group (in GCO2 sample), the result is completely reasonable. Moreover, keeping in mind the results obtained by other methods, it can be concluded that the observed mass loss is as high as expected. The mass content of phosphorus in the material determined by EDX analysis was equal to about 1.1%. Assuming that the phosphorus is only in the form of a bis-phosphonic group, and taking into consideration the structure of a bis-phosphonic group, the weight loss caused by the decomposition of all bis-phosphonic groups in GPhos should be approximately 3.8% of initial mass. It can be clearly seen in a TG curve for the GPhos sample that the mass loss in the temperature range 200–600 °C is absolutely in agreement with the estimated value. 

In contrast, the TG curve for GM sample is visibly different (SI, Figure S3), with a well-pronounced step in the 40–200 °C range and the highest total mass loss among all analyzed samples. As was discussed in Section 3.2, the graphite ball-milling in air atmosphere led to partial degradation of the material, making it less thermally stable.

### 3.7. BET Analysis

SSA of the GM, GCO2 and GPhos samples were determined from nitrogen adsorption–desorption isotherms (SI, Figure S4). For ball-milled graphite, the SSA was ca. 4 m^2^/g. As expected, ball-milling of graphite in presence of CO_2_ resulted in higher specific area (28 m^2^/g), which is mainly associated with graphite flakes exfoliation. Importantly, after reaction with PCl_3_, there is no obvious change in SSA. The slight decrease of surface area to 25 m^2^/g for GPhos could be connected with the partial removal of the smallest particles of GPhos during washing after the performed reaction. The smallest and the most exfoliated graphene platelets show the highest surface area and the highest disspersability. Therefore, they could be more easily removed from the sample during the washing procedure, decreasing the mean surface area of the analysed sample. For other samples, which is GM and GCO2, no washing procedure was necessary.

### 3.8. Adsorption Efficiency and Selectivity

**Figure 7:**
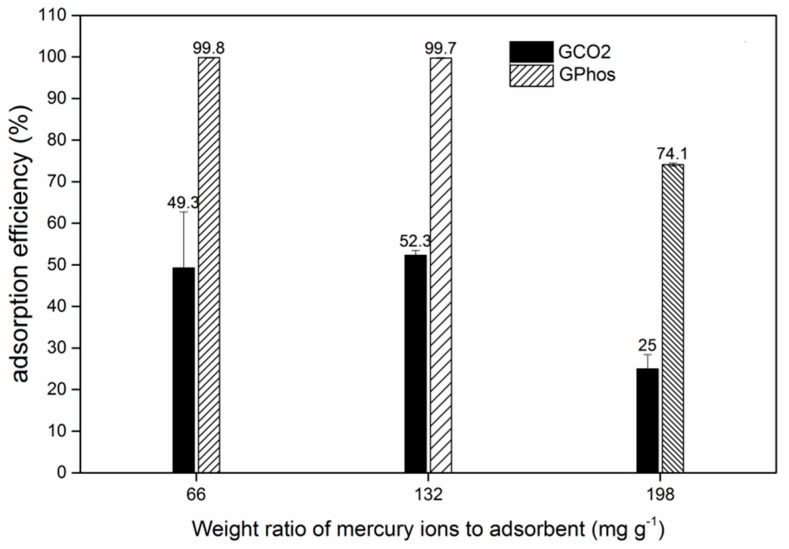
Adsorption efficiency of mercury ions for the GCO2 and GPhos sample.

In order to investigate the influence of functionalization on adsorption efficiency, adsorption studies have been performed using GCO2 and GPhos as adsorbents. Studies were carried out at different concentrations of adsorbate and results are presented with respect to the weight ratio of Hg^2+^ ions to adsorbent (Figure 7). In all cases, the GPhos showed a much higher adsorption efficiency than GCO2. Taking into account that SSA of GCO2 and GPhos were almost the same, it can be concluded that increasing in Hg^2+^ adsorption efficiency of GPhos is due to the presence of phosphonic groups. The difference between adsorption efficiency increases with growing mercury to adsorbent ratio and, for two studied Hg/adsorbent ratios, the removal efficiency exceeded 99% for the GPhos sample.

**Figure 8:**
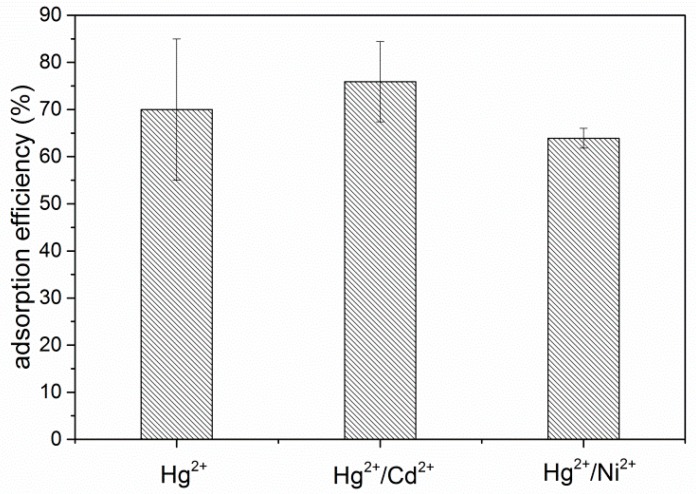
Influence of the presence of cadmium (1 mg/L) and nickel ions (1 mg/L) on adsorption efficiency of mercury.

Furthermore, adsorption efficiency of Hg^2+^ ions in the binary system was also investigated. Measurements were conducted in the presence of additional bivalent metal ions: cadmium and nickel, whose initial concentrations were set on the same level as the concentration of Hg^2+^ ions (1 mg/L). It was found that coexisting Cd^2+^ and Ni^2+^ ions have no significant impact on Hg^2+^ adsorption efficiency. As presented in Figure 8, the uptake of Hg^2+^ ions in binary systems was comparable to the uptake observed for bare mercury ion solution (70 +/− 6% in all three cases).

From Pearson’s theory, the relative affinities of metals for different ligands follow the general pattern: hard metals interact more strongly with hard ligands and soft metals with soft ligands. The phosphonic acid and Hg^2+^ are soft base and acid, respectively. The Ni^2+^ and Cd^2+^ ions are borderline examples, that is, they are neither soft nor hard acids. It means that the complexation is more favorable for mercury ions. This could be the reason why the removal of mercury is independent from the presence of other ions. The second factor that may have an influence on the selectivity is the reaction kinetics. The adsorption of mercury could be the fastest among analyzed ions; the equilibrium is reached in the shortest time, leading to more efficient removal of these ions. Similar results for graphene based adsorbents were obtained by others [50,51,52].

### 3.9. Langmuir and Freundlich Isotherm Model

**Figure 9:**
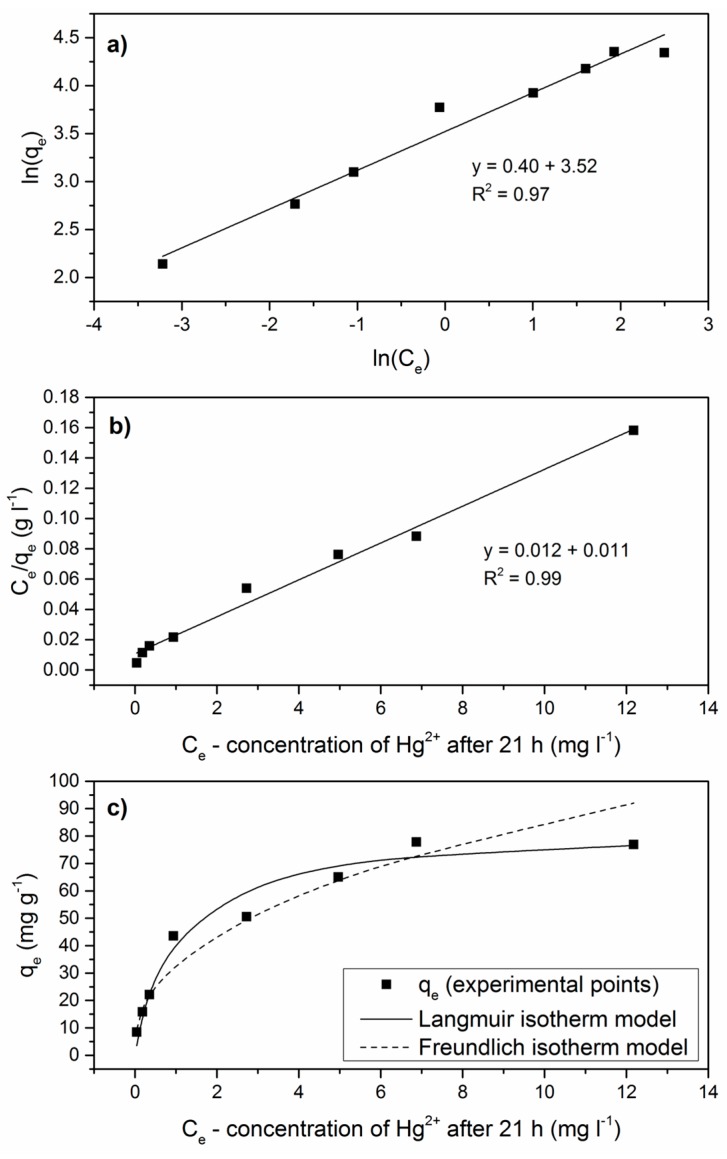
Adsorption isotherm models (**a**) Freundlich isotherm model; (**b**) Langmuir isotherm model; (**c**) experimental points fitted with Langmuir and Freundlich isotherm models.

The influence of concentration of mercury ions to adsorption efficiency was investigated. The experimental data were fitted to the Langmuir and Freundlich adsorption isotherm models (Figure 9). Obtained results revealed that the Langmuir model provides a better fit than the Freundlich model (0.99 < R^2^ < 0.97). This indicates the homogenous distribution of active sites on the GPhos surface and a monolayer coverage. Langmuir maximum capacity q_m_ and constant b were equal to 82.2 mg/g, and 1.13 L/mg, respectively. Moreover, the R_L_ parameter has also been calculated and its values were in the range 0–1, confirming that the adsorption process of Hg^2+^ ions onto GPhos surface is favorable.

High surface area and hydrophilic character make the graphene oxide an interesting adsorbent. Therefore, there is some literature concerning its metal ions’ adsorption abilities, including mercury cations [50,51,52,53,54,55]. The summary of reported data is presented in Table S1. However, the synthesis of GO is carried out under harsh conditions and the purification of GO produces a huge amount of water contaminated with manganese or chromium compounds, and others, depending on the applied protocol. Moreover, pristine GO does not show high sorption abilities towards Hg (23 mg/g according to [53]) and different functionalization approaches are implemented. In the most frequently performed procedure, ester or amide bonds are created between oxygen groups in GO and attached moiety, which are unfortunately prone to hydrolysis. Consequently, it is possible that, during sorption studies, the adsorbent is partially destroyed and may contaminate the purified medium. Taking this into account, the adsorption properties of our material, which are not the highest among reported values, are still significant. The material is obtained by a solvent-free, simple and low cost method and the functionalization with phosphonic groups creates a stable C-P bond, which does not hydrolyze. Therefore, the adsorbent reported by us is the golden mean between the expected properties and simplicity and costs of its manufacturing.

### 3.10. Adsorption Kinetics Studies

**Figure 10:**
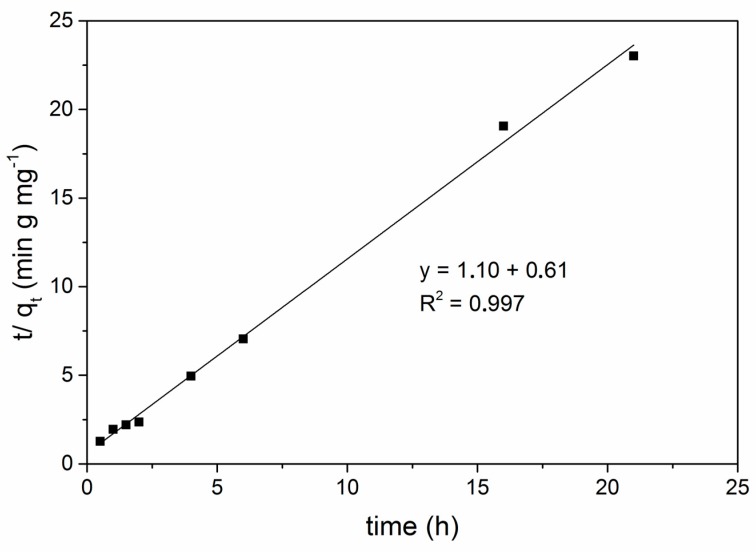
Adsorption kinetics studies pseudo-II order equation.

An appropriate kinetic model can quantify the changes in analyte adsorption with time, which is important for commercial usage of adsorbent. Two kinetic models, i.e., the pseudo first-order and pseudo second-order models were applied to explain Hg^2+^ion adsorption behavior by a studied adsorbent. Analyzing the results, it was found out that the pseudo-first model is not sufficiently good for describing the process. The plot presented in Supplementary (Figure S5), clearly demonstrates the lack of linearity. As can be seen in Figure 10, a much better fit was obtained for a pseudo-second order equation and the correlation coefficient is greater than 0.997. The parameters k_2_ and h for a pseudo-second order model were equal to 1.98 g/h∙mg and 1.65 mg/g∙h, respectively. The obtained results suggest that this sorption system gave the best correlation data for the pseudo-second order model, based on the assumption that the rate-limiting step may be chemical sorption or chemisorption involving valence forces through sharing or exchange of electrons between sorbent and sorbate. In other words, the efficiency of the sorption system stays in correlation with the availability of adsorption sites on the surface of adsorbent rather than adsorbate concentration in bulk solution. It was also shown in the literature that adsorption systems based on carbonaceous materials most frequently follow the pseudo-second order kinetics.

## 4. Conclusions

A simple and economically friendly method for graphene derivatives manufacturing was presented. The ball-milling approach, together with solvent-free chemical transformation, is operationally convenient and environmentally benign. Contrary to graphene oxide synthesis, in the method proposed by us, the waste production is negligible. The obtained material was fully characterized using spectroscopy, electron microscopy and thermal analysis. All results confirmed the expected structure. The obtained graphene derivative with phosphorus bearing groups was proposed as an efficient adsorbent for mercury ions removal from water. The removal efficiency exceeded 99% for the functionalized adsorbent. Experimental data fit well to the Langmuir (R^2^ = 0.99) isotherm model. Maximum adsorption capacity *q_s_* and energy constant of the adsorption capacity *b* were calculated and were equal to 82.2 mg/g and 1.13 L/mg, respectively. The adsorption kinetics studies revealed that the adsorption kinetics followed the pseudo-second order kinetic model, which is frequently observed for carbonaceous materials. Moreover, the presence of others’ bivalent ions like cadmium or nickel ions have no significant impact on adsorption efficiency of mercury ions. The most important advantages of investigated material are high adsorption efficiency and simple and low-cost production.

## Figures and Tables

**Table 1:** Analysis of Raman spectra for GM, GCO2, GPhos.

Sample	D’-G_app_ (cm^−1^)	D	G (cm^−1^)	I_D_/I_G_	D’ *_fwhm_* (cm^−1^)	2D’-G_app_ (cm^−1^)
GM	40	1343	1571	1.06	31	1354
GCO2	36	1351	1582	1.00	21	1365
GPhos	36	1351	1581	0.91	18	1368

**Table 2:** Elemental composition of GCO2 and GPhos calculated from X-ray Photoelectron Spectrocopy survey spectra.

Sample	Atomic Content (%)		
	C	O	P
GCO2	94.6	5.4	-
GPhos	85.4	14.2	0.4

## Supplementary Materials

The following are available online at https://www.mdpi.com/2079-4991/9/4/485/s1, Figure S1. XPS survey spectrum of GCO2; Figure S2. XPS survey spectrum of GPhos; Figure S3. TG curve for GM sample (ball-milled graphite); Figure S4. Nitrogen adsorption–desorption isotherms for analyzed samples; Figure S5. Plotted data revealing, that pseudo-first order model is not suitable in this case. Table S1. Comparison of Hg(II) adsorption capacity of graphene based adsorbents.

## References

[1] Geim A.K. (2009). Graphene: Status and prospects. Science.

[2] Neto A.C., Guinea F., Peres N.M., Novoselov K.S., Geim A.K. (2009). The electronic properties of graphene. Rev. Mod. Phys..

[3] Soldano C., Mahmood A., Dujardin E. (2010). Production, properties and potential of graphene. Carbon.

[4] Novoselov K.S., Fal V.I., Colombo L., Gellert P.R., Schwab M.G., Kim K. (2012). A roadmap for graphene. Nature.

[5] Iwan A., Malinowski M., Pasciak G. (2015). Polymer fuel cell components modified by graphene: Electrodes, electrolytes and bipolar plates. Renew. Sustain. Energy Rev..

[6] Wang X., Zhi L., Müllen K. (2008). Transparent, conductive graphene electrodes for dye-sensitized solar cells. Nano Lett..

[7] Han T.H., Kim H., Kwon S.J., Lee T.W. (2017). Graphene-based flexible electronic devices. Mater. Sci. Eng. R. Rep..

[8] Justino C.I., Gomes A.R., Freitas A.C., Duarte A.C., Rocha-Santos T.A. (2017). Graphene based sensors and biosensors. TrAC.

[9] Bonaccorso F., Sun Z., Hasan T., Ferrari A.C. (2010). Graphene photonics and optoelectronics. Nat. Photonics.

[10] Mohan V.B., Lau K.T., Hui D., Bhattacharyya D. (2018). Graphene-based materials and their composites: A review on production, applications and product limitations. Compos. Part B Eng..

[11] Georgakilas V., Otyepka M., Bourlinos A.B., Chandra V., Kim N., Kemp K.C., Hobza P., Zboril R., Kim K.S. (2012). Functionalization of graphene: Covalent and non-covalent approaches, derivatives and applications. Chem. Rev..

[12] Kuila T., Bose S., Mishra A.K., Khanra P., Kim N.H., Lee J.H. (2012). Chemical functionalization of graphene and its applications. Prog. Mater. Sci..

[13] Boissezon R., Muller J., Beaugeard V., Monge S., Robin J.J. (2014). Organophosphonates as anchoring agents onto metal oxide-based materials: Synthesis and applications. RSC Adv..

[14] Zhao R., Rupper P., Gaan S. (2017). Recent development in phosphonic acid-based organic coatings on aluminum. Coatings.

[15] Vahabi H., Longuet C., Ferry L., David G., Robin J.J., Lopez-Cuesta J.M. (2012). Effect of aminobisphosphonated copolymer on the thermal stability and flammability of poly (methyl methacrylate). Polym. Int..

[16] Opwis K., Wego A., Bahners T., Schollmeyer E. (2011). Permanent flame retardant finishing of textile materials by a photochemical immobilization of vinyl phosphonic acid. Polym. Degrad. Stab..

[17] Choong C., Griffiths J.P., Moloney M.G., Triffitt J., Swallow D. (2009). Direct introduction of phosphonate by the surface modification of polymers enhances biocompatibility. React. Funct. Polym..

[18] Liu M., Chen S., Zhao X., Ye Y., Li J., Zhu Q., Zhao B., Zhao W., Huang X., Shen J. (2013). Biocompatible phosphonic acid-functionalized silica nanoparticles for sensitive detection of hypoxanthine in real samples. Talanta.

[19] Schull T.L., Knight D.A. (2005). Organometallic phosphonic acids: Synthesis and coordination chemistry. Coord. Chem. Rev..

[20] Di Credico B., Redaelli M., Bellardita M., Calamante M., Cepek C., Cobani E., D’Arienzo M., Evangelisti C., Marelli M., Moret M. (2018). Step-by-step growth of HKUST-1 on functionalized TiO_2_ surface: An efficient material for CO_2_ capture and solar photoreduction. Catalysts.

[21] Żelechowska K., Prześniak-Welenc M., Łapiński M., Kondratowicz I., Miruszewski T. (2017). Fully scalable one-pot method for the production of phosphonic graphene derivatives. Beilstein J. Nanotechnol..

[22] Some S., Shackery I., Kim S.J., Jun S.C. (2015). Phosphorus-doped graphene oxide layer as a highly efficient flame retardant. Chem. Eur. J..

[23] Kim M.J., Jeon I.Y., Seo J.M., Dai L., Baek J.B. (2014). Graphene phosphonic acid as an efficient flame retardant. ACS Nano.

[24] Zakeri M., Abouzari-lotf E., Miyake M., Mehdipour-Ataei S., Shameli K. (2019). Phosphoric acid functionalized graphene oxide: A highly dispersible carbon-based nanocatalyst for the green synthesis of bio-active pyrazoles. Arab. J. Chem..

[25] He L., Zhao Y., Xing L., Liu P., Wang Z., Zhang Y., Liu X. (2017). Preparation of phosphonic acid functionalized graphene oxide-modified aluminum powder with enhanced anticorrosive properties. Appl. Surf. Sci..

[26] Jeon I.Y., Shin Y.R., Sohn G.J., Choi H.J., Bae S.Y., Mahmood J., Dai L. (2012). Edge-carboxylated graphene nanosheets via ball milling. Proc. Natl. Acad. Sci. USA.

[27] Jeon I.Y., Bae S.Y., Seo J.M., Baek J.B. (2015). Scalable Production of Edge-Functionalized Graphene Nanoplatelets via Mechanochemical Ball-Milling. Adv. Fun. Mater..

[28] Fan X., Chang D.W., Chen X., Baek J.B., Dai L. (2016). Functionalized graphene nanoplatelets from ball milling for energy applications. Curr. Opin. Chem. Eng..

[29] Yan L., Lin M., Zeng C., Chen Z., Zhang S., Zhao X., Guo M. (2012). Electroactive and biocompatible hydroxyl-functionalized graphene by ball milling. J. Mater. Chem..

[30] Leon V., Quintana M., Herrero M.A., Fierro J.L., de la Hoz A., Prato M., Vazquez E. (2011). Few-layer graphenes from ball-milling of graphite with melamine. Chem. Commun..

[31] Mahmoud A.E.D., Stolle A., Stelter M. (2018). Sustainable Synthesis of High-Surface-Area Graphite Oxide via DryBall Milling. ACS Sustain. Chem. Eng..

[32] Ramesha G.K., Kumara A.V., Muralidhara H.B., Sampath S. (2011). Graphene and graphene oxide as effective adsorbents toward anionic and cationic dyes. J. Colloid Interfce Sci..

[33] Kyzas G.Z., Deliyanni E.A., Matis K.A. (2014). Graphene oxide and its application as an adsorbent for wastewater treatment. J. Chem. Technol. Biotechnol..

[34] Kondratwicz I., Żelechowska K. (2017). Graphene Oxide as Mine of Knowledge: Using Graphene Oxide to Teach Undergraduate Students Core Chemistry and Nanotechnology Concept. J. Chem. Educ..

[35] Marcano D.C., Kosynkin D.V., Berlin J.M., Sinitskii A., Sun Z., Slesarev A., Alemany L.B., Lu W., Tour J.M. (2010). Improved Synthesis of Graphene Oxide. ACS Nano.

[36] Wojtoniszak M., Mijowska E. (2012). Controlled oxidation of graphite to graphene oxide with novel oxidants in a bulk scale. J. Nanopart. Res..

[37] Chen J., Yao B., Li C., Shi G. (2013). An improved Hummers method for eco-friendly synthesis of graphene oxide. Carbon.

[38] Romanenko V.D., Kukhar V.P. (2012). 1-Amino-1,1-bisphosphonates-Fundamental Syntheses and New Developments. ChemInform.

[39] Kaabak L.V., Kuz’mina N.E., Khudenko A.V., Tomilov A.P. (2006). Improved Synthesis of 1-Aminoethylidenediphosphonic Acid. Russ. J. Gen. Chem..

[40] Lecouvey M., Leroux Y. (2000). Synthesis of 1-Hydroxy-1,1-bisphosphonates. Heteroatom Chem..

[41] Chmielewska E., Kafarski P. (2016). Synthetic Procedures Leadingtowards Aminobisphosphonates. Molecules.

[42] Kieczykowski G.R., Jobson R.B., Melillo D.G., Reinhold D.F., Grenda V.J., Shinkai I. (1995). Preparation of (4-Amino-1-Hydroxybutylidene)bisphosphonic Acid Sodium Salt, MK 217(Alendronate Sodium). An Improved Procedure for the Preparation of 1-Hydroxy-1,1-bisphosphonic Acids. J. Org. Chem..

[43] Simonin J.P. (2016). On the comparison of pseudo-first order and pseudo-second order rate laws in the modeling of adsorption kinetics. Chem. Eng. J..

[44] Do D.D. (1998). Adsorption Analysis: Equilibria and Kinetics.

[45] Żelechowska K., Sobota D., Cieślik B., Prześniak-Welenc M., Łapiński M., Biernat J.F. (2018). Bis-phosphonated carbon nanotubes: One pot synthesis and their application as efficient adsorbent of mercury. Fuller. Nanotubes Carbon Nanostruct..

[46] King A.A., Davies B.R., Noorbehesht N., Newman P., Church T.L., Harris A.T., Minett A.I. (2016). A New Raman Metric for the Characterisation of Graphene oxide and its Derivatives. Sci. Rep..

[47] Pei S., Zhao J., Du J., Ren W., Cheng H.M. (2010). Direct reduction of graphene oxide films into highly conductive and flexible graphene films by hydrohalic acids. Carbon N. Y..

[48] Stobinski L., Lesiak B., Malolepszy A., Mazurkiewicz M., Mierzwa B., Zemek J., Bieloshapka I. (2014). Graphene oxide and reduced graphene oxide studied by the XRD, TEM and electron spectroscopy methods. J. Electron Spectrosc. Relat. Phenomena.

[49] Düngen P., Schlögl R., Heumann S. (2018). Non-linear thermogravimetric mass spectrometry of carbon materials providing direct speciation separation of oxygen functional groups. Carbon.

[50] Kumar A.S., Jiang S.J. (2015). Preparation and characterization of exfoliated graphene oxide-L-cystine as an effective adsorbent of Hg(II) adsorption. RSC Adv..

[51] Kumar A.S., Jianga S.-J., Tseng W.-L. (2016). Facile synthesis and characterization of thiol-functionalized graphene oxide as effective adsorbent for Hg(II). J. Environ. Chem. Eng..

[52] Henriques B., Goncalves G., Emami N., Pereira E., Vila M., Marques P.A.A.P. (2016). Optimized graphene oxide foam with enhanced performance and high selectivity for mercury removal from water. J. Hazard. Mater..

[53] Awad S.F., AbouZied K.M., El-Maaty W.M.A., El-Wakil A.M., El-Shall M.S. Effective removal of mercury(II) from aqueous solutions by chemically modified graphene oxide nanosheets. Arab. J. Chem..

[54] Gao W., Majumder M., Alemany L.B., Narayanan T.N., Ibarra M.A., Pradhan B.K., Ajayan P.M. (2011). Engineered Graphite Oxide Materials for Application in Water Purification. ACS Appl. Mater. Interfaces.

[55] Nuengmatcha P., Mahachai R., Chanthai S. (2015). Adsorption of Functionalized Thiol-Graphene Oxide for Removal of Mercury from Aqueous Solution. Asian J. Chem..

