# Supplementary material for: Solvent-Free Synthesis of Phosphonic Graphene Derivative and Its Application in Mercury Ions Adsorption
Source: Nanomaterials (Basel). 2019 Mar 27;9(4):485. doi: 10.3390/nano9040485 (PMC6523945; doi:10.3390/nano9040485)
Supplement: Supplementary file 1 [file nanomaterials-09-00485-s001.pdf]

Supplementary material

Solvent-free synthesis of phosphonic graphene derivative and its application in mercury ions adsorption

Robert Olszewski [1,†], Małgorzata Nadolska [1,†], Marcin Łapiński [1], Marta Prześniak-Welenc [1], Bartłomiej Michał Cieślik [2] and Kamila Żelechowska [1,*]

1 Department of Solid State Physics, Faculty of Applied Physics and Mathematics, Gdansk University of Technology, Narutowicza 11/12, 80-233 Gdansk, Poland; robercik.olszewski@gmail.com (R.O.); malgorzata.nadolska@pg.edu.pl (M.N.); marcin.lapinski@pg.edu.pl (M.Ł.); marta.welenc@pg.edu.pl (M.P.-W.)
2 Faculty of Chemistry, Department of Analytical Chemistry, Gdansk University of Technology, Narutowicza St. 11/12, 80-233 Gdansk, Poland; cieslik1988@wp.pl
* Correspondence: kamila.zelechowska@pg.edu.pl; Tel.: +48-58-348-66-16
† These authors contributed equally to this work

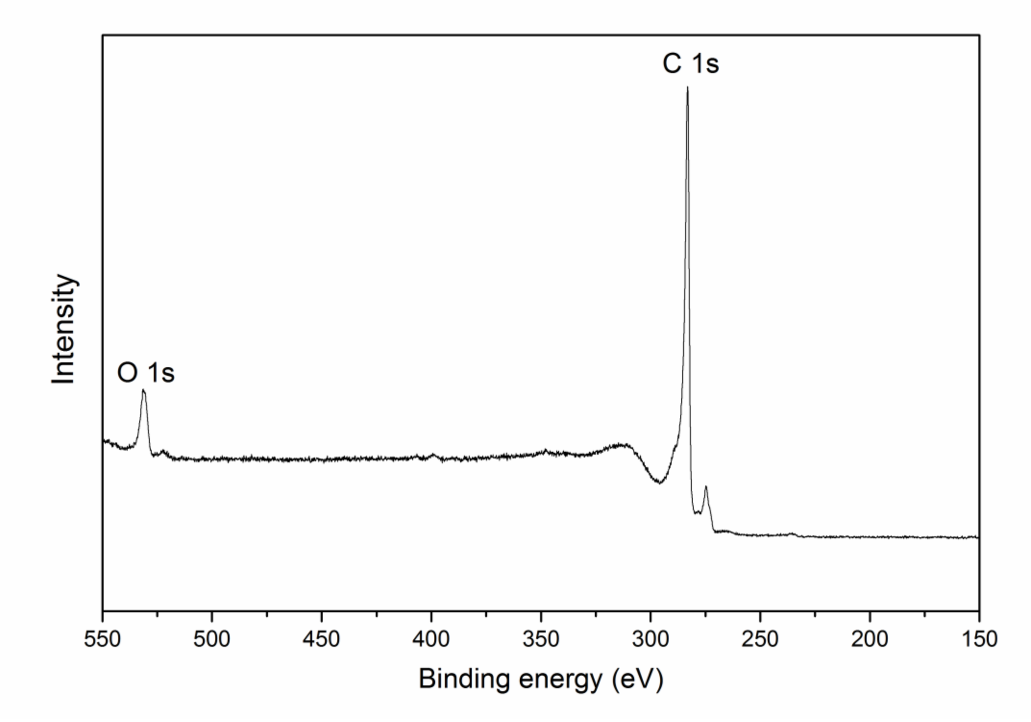

Figure S1. X-ray Photoelectron Spectroscopy survey spectrum of GCO2

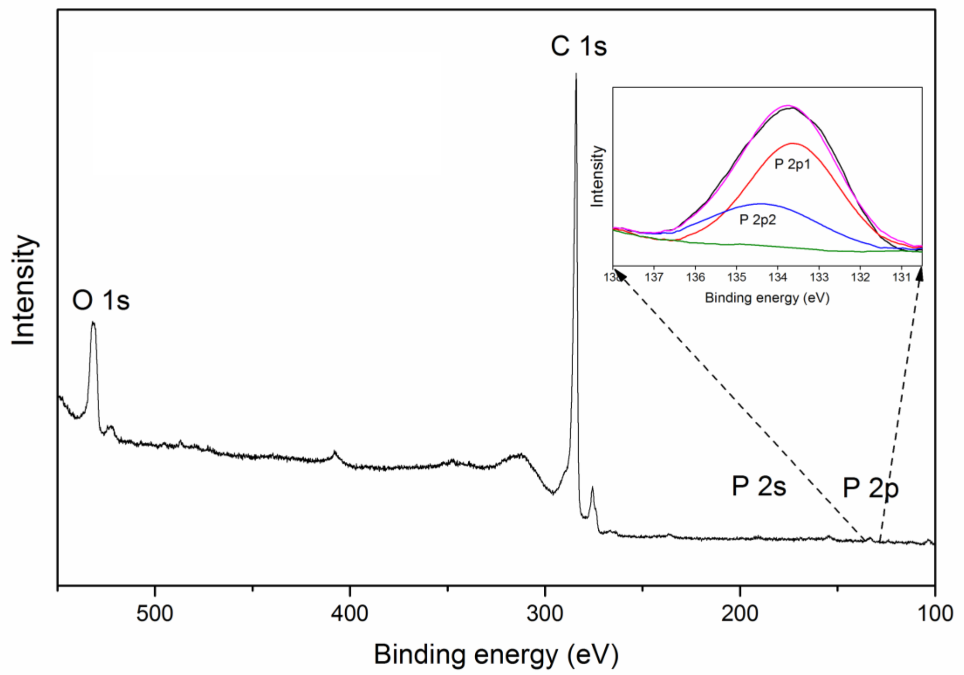

Figure S2. X-ray Photoelectron Spectroscopy survey spectrum of GPhos

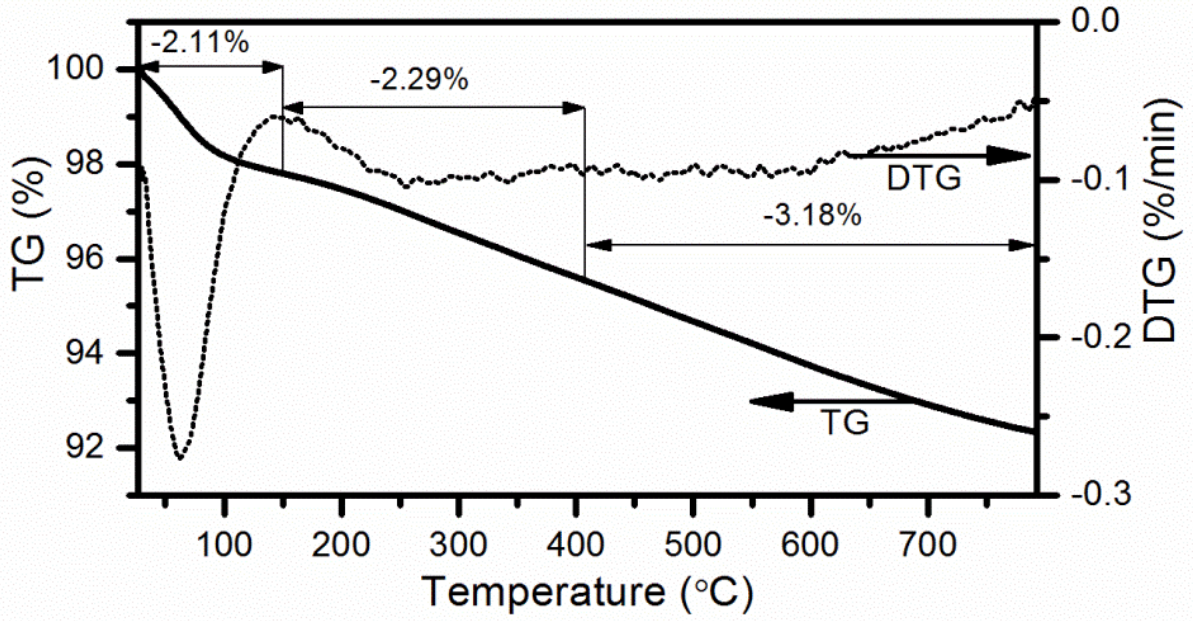

Figure S3. Thermal gravimetric curve for GM sample (ball-milled graphite)

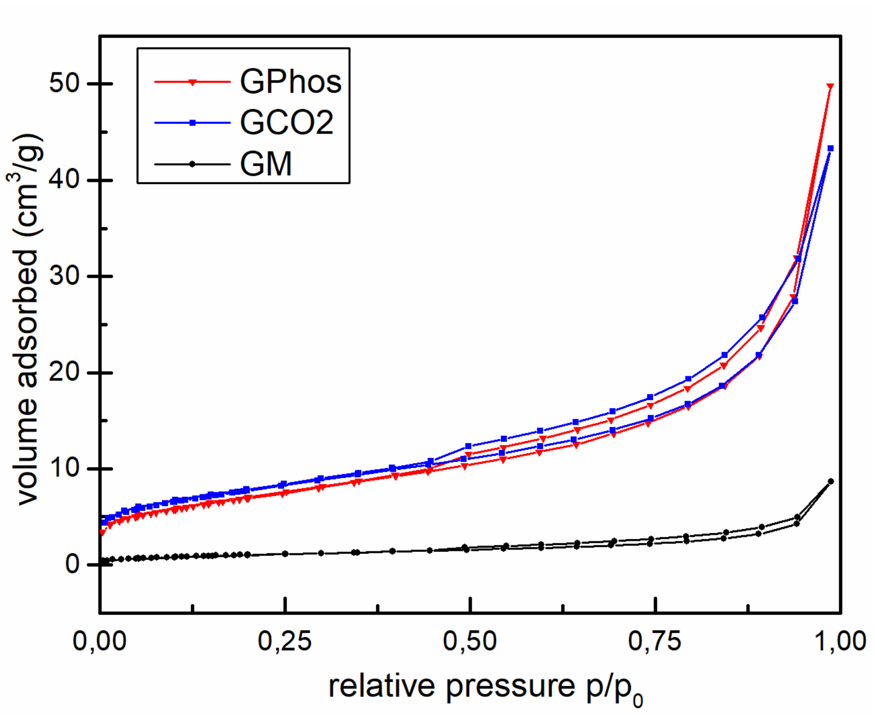

Figure S4.Nitrogen adsorption–desorption isotherms for analyzed samples

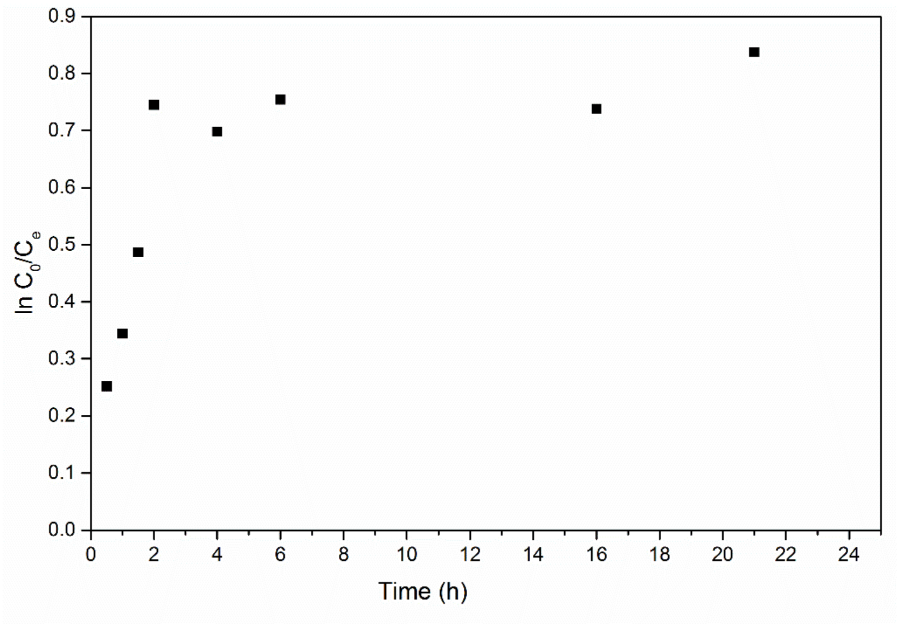

Figure S5. Plotted data revealing, that pseudo-first order model is not suitable in this case

Table S1. Comparison of Hg(II) adsorption capacity of graphene based adsorbents

No	Adsorbent	Adsorption capacity mg/g	pros and cons	Ref.
1.	L-cysteine functionalized graphene oxide (GO)	79.36	bonds prone to hydrolysis, cysteine is safe and available	[50]
2.	GO functionalized with mercaptobenzothiazole (MBT)	107.52	MBT is possible human carcinogen	[51]
3.	GO sponge with nitrogen functional groups	35	prepared by high temperature and pressure procedure, hydrophobic character	[52]
4.	GO	23.0	hydrophilic; difficulties with its removal from water	[53]
5.	acetic acid functionalized GO	128.2	simple functionalization protocol; bonds prone to hydrolysis	[53]
6.	imino-diacetic acid functionalized GO	247.5	bonds prone to hydrolysis; multi-step reaction	[53]
7.	thiophenol-decorated reduced GO	30	aminothiophenol used in the reaction is highly toxic	[54]
8.	thiophenol-functionalized GO	200	aminothiophenol used in the reaction is highly toxic	[54]
9.	3-mercaptopropyl-trimethoxysilane functionalized GO	80.65	expensive and water sensitive reagent	[55]
10.	phosphonic derivative of graphene	82.2	solvent-free low-cost synthesis, no-wastes produced; cheap reagents, non-hydrolysable C-P bond	this work
